# Comparative Cuticle Development Reveals Taller Sporophytes Are Covered by Thicker Calyptra Cuticles in Mosses

**DOI:** 10.3389/fpls.2016.00832

**Published:** 2016-06-14

**Authors:** Jessica M. Budke, Bernard Goffinet

**Affiliations:** ^1^Department of Plant Biology, University of California, Davis, DavisCA, USA; ^2^Department of Ecology and Evolutionary Biology, University of Connecticut, StorrsCT, USA

**Keywords:** bryophyte, calyptra, comparative development, cuticle, dehydration stress, electron microscopy, maternal effects, sporophyte protection

## Abstract

The calyptra is a maternal structure that protects the sporophyte offspring from dehydration, and positively impacts sporophyte survival and fitness in mosses. We explore the relationship between cuticle protection and sporophyte height as a proxy for dehydration stress in Funariaceae species with sporophytes across a range of sizes. Calyptrae and sporophytes from four species were collected from laboratory-grown populations at two developmental stages. Tissues were embedded, sectioned, and examined using transmission electron microscopy. Cuticle thickness was measured from three epidermal cells per organ for each individual and compared statistically. All four species have cuticles consisting of a cuticle proper and a cuticular layer on the calyptra and sporophyte at both developmental stages. Across species, shorter sporophytes are associated with smaller calyptra and thinner calyptra cuticles, whereas taller sporophytes are associated with larger calyptra and thicker calyptra cuticles. Independent of size, young sporophytes have a thin cuticle that thickens later during development, while calyptrae have a mature cuticle produced early during development that persists throughout development. This study adds to our knowledge of maternal effects influencing offspring survival in plants. Released from the pressures to invest in protection for their sporophyte offspring, maternal resources can be allocated to other processes that support sporophyte reproductive success. Using a comparative developmental framework enables us to broaden our understanding of cuticle development across species and provides structural evidence supporting the waterproofing role of the moss calyptra.

## Introduction

The ability to decrease water loss was critical for the evolution and survival of plants in terrestrial environments (Graham, 1993). On the aerial organs of plants, water loss is decreased by the cuticle, a modified cell wall region consisting of polysaccharides and a polymer matrix of cutins/cutans embedded with waxes and phenolics, in addition to waxes deposited on the outer surface of the matrix (Domínguez et al., 2011; Guzmán et al., 2014). The cuticle occurs on all land plants, including mosses (Busta et al., 2016), and it is important for protection from ultraviolet (UV) radiation (Krauss et al., 1997; Holmes and Keiller, 2002; Pfündel et al., 2006), self-cleaning of photosynthetic surfaces (Barthlott and Neinhuis, 1997), and prevention of pathogen attacks (Campbell et al., 1980). Cuticles occur in all lineages of land plants and play critical roles even in the earliest diverging lineages (i.e., liverworts, hornworts, and mosses). On the vegetative gametophytes of these plants, cuticles create water-free regions on the external plant body that facilitate gas exchange, which occurs directly through the epidermal cell walls, given the absence of stomata (Schönherr and Ziegler, 1975; Thomas et al., 1996). The cuticle also maintains internal hydration in mosses for both the gametophytes of endohydric taxa (Proctor, 1979) and for the relatively long-lived sporophytes (Budke et al., 2013).

Moss sporophytes begin development within the female reproductive organs (archegonia) and are protected by the surrounding leaves of the maternal gametophyte. As development progresses, the sporophyte increases in height and its exposure to dehydration stress is magnified as it emerges from the leaves of the maternal gametophyte and the protective influence of the laminar boundary layer (Proctor, 1980, 1982; Rice et al., 2001; Rice and Schneider, 2004). Young moss sporophytes have a thin cuticle that structurally is unable to protect them from dehydration (French and Paolillo, 1975; Budke et al., 2012). Protection for the dehydration-sensitive sporophyte apex during the critical developmental stage of stalk-building is provided by the calyptra, a cap of maternal gametophyte tissue that has a thick, multilayered cuticle (Budke et al., 2011). This cuticle-covered cap forms early in development and protects the moss sporophyte apex until the sporophyte later develops a thicker cuticle, during capsule maturation (Budke et al., 2012). The calyptra cuticle is functionally important for sporophyte survival, development, and fitness (Budke et al., 2013).

Sporophyte morphology is highly variable across the 12,500 species of mosses with sporophyte heights ranging from over 9 cm tall in *Polytrichum* Hedw. (Smith Merrill, 2007) to less than 1 mm in *Micromitrium* Austin (Goffinet et al., 2011). Sporophyte height can vary within a family or genus, potentially accompanying a shift to drier or seasonally moist habitats or in the other extreme a shift to aquatic habitats (Vitt, 1981). Within the Funariaceae Schwägr., a wide range of sporophyte sizes is represented (Fife, 1982). Ranging from taxa with a tall stalk elevating the capsule high above the maternal plant (e.g., *Funaria hygrometrica* Hedw.) to species that essentially lack a stalk and thus have a capsule, which at maturity is immersed among the leaves originally surrounding the female sex organs [e.g., *Aphanorrhegma serratum* (Hook and Wilson) Sull.]. This wide diversity among closely related species makes the Funariaceae an ideal system for comparative studies of the sporophyte and its cuticle.

Growing under equivalent ecological conditions, shorter sporophytes would be anticipated to experience lower levels of dehydration stress compared to taller sporophytes due to the closely surrounding leaves and the protection afforded by the laminar boundary layer of the adjacent substrate (Proctor, 1980, 1982; Rice et al., 2001; Rice and Schneider, 2004). Released from the selective pressures of dehydration stress, the role of the cuticle in protection of the sporophyte may become unnecessary. Thus, retention of a thick cuticle in taxa with short sporophytes may point toward an alternative functional importance, such as protection from UV radiation (Krauss et al., 1997; Holmes and Keiller, 2002; Pfündel et al., 2006). The cuticle is a costly resource investment (Poorter and Villar, 1997). Decreasing the investment in this protective layer by either the maternal plant (i.e., the calyptra) or the offspring sporophyte frees up resources to devote to other processes that enhance fitness and thereby ultimately reproductive success, such as spore production.

In this study, we explore the relationship between cuticle thickness and sporophyte height as a proxy for dehydration stress in taxa with sporophytes across a range of sizes. We acknowledge that studies of vascular plant leaves and fruits have not confirmed Fick’s first law, that states cuticular permeability should be related to the thickness of the cuticular membrane (Norris, 1974; Riederer and Schreiber, 2001). The quantities of waxes and/or cutins have also not been shown to predict cuticular permeability (Riederer and Schreiber, 2001; Yeats and Rose, 2013). Instead, the ratios between waxes classes, rather than the overall quantities, have been shown to correlate with cuticular permeability (Parsons et al., 2012). Analyzing the structural variation in bryophyte cuticles comparatively across taxa and development is the first step in exploring Fick’s first law in bryophytes. Bryophytes have cuticles that are orders of magnitude thinner than vascular plants (Jeffree, 2006) and are separated by at least 420 million years of evolution from their most recent common ancestors (Clarke et al., 2011). Alternative relationships may emerge for bryophytes, potentially including a correlation between cuticle thickness and permeability, which currently remains to be tested.

We know that the maternal plant invests in a relatively thick calyptra cuticle that protects young sporophytes from the stress of dehydration in *F. hygrometrica* (Budke et al., 2011, 2013). First, we sought to confirm (i) that young sporophytes of all species have a very thin cuticle during early development and (ii) that sporophytes develop a thick cuticle later in their development. Given the cuticle developmental patterns in (i) and (ii) are confirmed, we hypothesize (iii) that the maternal investment in the calyptra cuticle would correlate with sporophyte height: the calyptra cuticle will be thinner for species with shorter sporophytes and thicker for species with taller sporophytes. We hypothesize that this investment in a calyptra cuticle will occur early and persist throughout sporophyte development. These hypotheses are based on shorter sporophytes likely encountering less dehydration stress during development, compared to taller sporophytes. Additionally we hypothesize (iv) that species with shorter sporophytes will have a thinner sporophyte cuticle compared to species with taller sporophytes. To address these hypotheses, we compared the development of the cuticle on the sporophyte and calyptra for four moss species in the Funariaceae that cover a range of sporophyte and calyptra sizes.

## Materials and Methods

### Study Taxa

**FIGURE 1:**
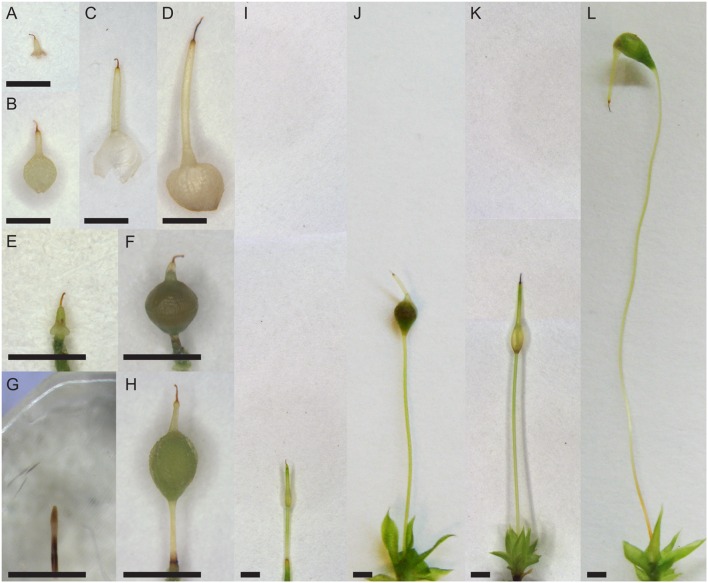
**Morphology of Funariaceae species.** **(A–D)** Older calyptrae. **(E,G,I,K)** Young sporophytes with apex covered by calyptrae, except **(G)** without calyptra. **(F,H,J,L)** Older sporophytes with calyptra on the top. **(A,E,F)** *Aphanorrhegma serratum*. **(B,G,H)** *Physcomitrellopsis africana*. **(C,I,J)** *Physcomitrium pyriforme*. **(D,K,L)** *Funaria hygrometrica*. Scale bars: 1 mm.

Mosses in the Funariaceae have very similar leafy gametophyte morphologies. The morphological diversity in this family lies in the maternal gametophyte calyptra and sporophyte (Fife, 1982). Four Funariaceae species with contrasting calyptra and sporophyte morphologies were cultured in the laboratory for this study. *Aphanorrhegma serratum* (NY Buck #49500) is very small with a sporophyte with a globose capsule that is less than 1 mm tall that remains immersed among the leaves of the maternal gametophyte even at maturity (**Figure 1F**). The calyptra of *A. serratum* is a small cap with several lobes at the base and overall is less than half a centimeter long (**Figures 1A,F**). *Physcomitrellopsis africana* Wagner and Broth. ex. Dixon (CONN Goffinet #10326) is a slightly larger moss with a sporophyte ending in an elliptic capsule that is less than 3 mm tall, and that remains relatively immersed among the leaves of the maternal gametophyte even at maturity (**Figure 1H**). The calyptra of *P. africana*, at less than 2 mm in length, has a short rostrum at the apex and an inflated base below that covers a majority of the sporophyte capsule at maturity (**Figures 1B,H**). *Physcomitrium pyriforme* (Hedw.) Hampe (CONN Goffinet #9276) has a relatively tall sporophyte (around 15 mm) with a globose-pyriforme capsule that is exerted above the maternal gametophyte at maturity (**Figure 1J**). The calyptra of *P. pyriforme* is 2–3 mm long, ends in a rostrum and is deeply split at the base in 2–4 lobes (**Figures 1C,J**). *Funaria hygrometrica* (CONN Budke #142) has a very tall sporophyte, which can reach heights of 80 mm elevating the asymmetrically curved capsule far above the maternal gametophyte at maturity (**Figure 1L**). The calyptra of *F. hygrometrica*, which can be 3–5 mm long, has a long rostrum and a wide inflated base that is split by a single slit (**Figures 1D,L**).

Leafy gametophytes were grown from spores of the original populations on a rich sandy loam soil mix at room temperature (approximately 22–25°C) under 16 h of daylight in PlantCon plant tissue culture containers (MP Biomedicals, Solon, OH, USA). *Aphanorrhegma serratum* produced gametangia and subsequently sporophytes after 2–3 months at room temperature. The other three taxa were grown at room temperature for 4 months, then cold treated at 10°C with 8 h daylight for 2 months to stimulate gametangial development. Deionized water was added to populations with gametangia, covering the leafy gametophytes, for 24 h to enhance fertilization. After the water was removed, plants remained in the cold growth chamber for one additional week. Populations were then placed at room temperature under 16 h of daylight to facilitate sporophyte development. Calyptra and sporophytes from each population were harvested at two developmental stages, young with a spear-shaped, unexpanded sporophyte, and older with an expanded sporangium containing spores, from individuals located toward the middle of the container to eliminate any potential edge effects (**Figure 1**).

### Transmission Electron Microscopy

To investigate cuticle ultrastructure the sporophyte apex, including the region of the expanded sporangium, and their associated calyptra were collected. The older sporangia and calyptrae were split longitudinally to facilitate fixation and infiltration. All tissues were immediately placed into fixative (1.5% glutaraldehyde, 1.5% formaldehyde in 0.05 M PIPES buffer, pH 7.0; Renzaglia et al., 1997) for 4–8 h under ambient conditions, then overnight under vacuum, for a total of 24 h of fixation. Tissues were rinsed in 0.05 M PIPES buffer twice for 20 min each and kept in buffer overnight at 4°C in the dark. Tissues were then rinsed in 0.05 M PIPES buffer once for 20 min. Osmium fixation (2.0% OsO4 in 0.05 M PIPES buffer, pH 7.0) was carried out for 2 h in the dark followed by three changes of distilled water for 30 min each. Dehydration was performed using a graded ethanol (EtOH) series of cold solutions with 30 min at each stage, with a two final rinses of 100% EtOH for 15 min each. After this step, tissues were embedded in Spurrs resin (Pelco, Redding, CA, USA) as outlined in Budke et al. (2011). Tissues were sectioned transversely using an Ultrotome III (LKB Produkter, Stockholm, Sweden) to 100 nm. Calyptrae were cut at the mid-rostrum region. Young sporophytes were cut within 1 mm of the apex, in a region that was beneath the calyptra prior to sampling. Older sporangia were sectioned at the widest point of the expanded capsule, approximately the capsule middle. Sections were placed on gold-coated copper slot grids with a layer of Formvar. All grids were stained in aqueous solutions (w/v) of 1.5% potassium permanganate (5 min), 2% uranyl acetate (5 min), then 2.5% lead citrate (2 min). Sections were examined and photographed using a Tecnai Biotwin (FEI Electron Optics, Eindhoven, Netherlands) transmission electron microscope at 80 kV accelerating voltage.

### Morphology

The length of five calyptrae and five sporophytes for each species at each developmental stage was measured from the pool of fixed specimens to determine the average sizes of the examined structures. Calyptra length was measured from the top of the narrow rostrum, if present, to the bottom edge of the inflated base. Sporophyte height was measured from the top of the apex or capsule, to the base of the seta, excluding the foot.

### Statistical Analyses

For both the calyptra and sporophyte, three epidermal cells equally spaced around the circumference were measured for thicknesses of the cuticle layers (cuticle proper – an electron lucent layer adjacent and exterior to the dark filaments of the cell wall; cuticular layer – an electron lucent layer visibly intermingled with dark filaments of the cell wall), at the middle of the periclinal cell walls. Also the cell wall thicknesses and lumen sizes for each of these cells were measured to determine when the organs have completed their development. All measurements were taken from digital images using the program ImageJ^1^. All data were analyzed and figures created using the program R 3.0.2 (R Development Core Team, 2013).

Differences between young and older developmental stages were assessed for each species. Sample variances were compared and paired *t*-tests were performed with an adjustment for unequal variances as needed. To assess differences across species, ANOVAs were used, followed by Tukey *post hoc* tests for significant ANOVAs (*P* < 0.05) to determine whether significant differences occurred between pairs of species. Sample variances were compared and paired *t*-tests were performed with an adjustment for unequal variances as needed. A simple linear regression model was used to determine whether sporophyte height, as a proxy for dehydration stress, is related to both calyptra length and cuticle thickness.

## Results

**FIGURE 2:**
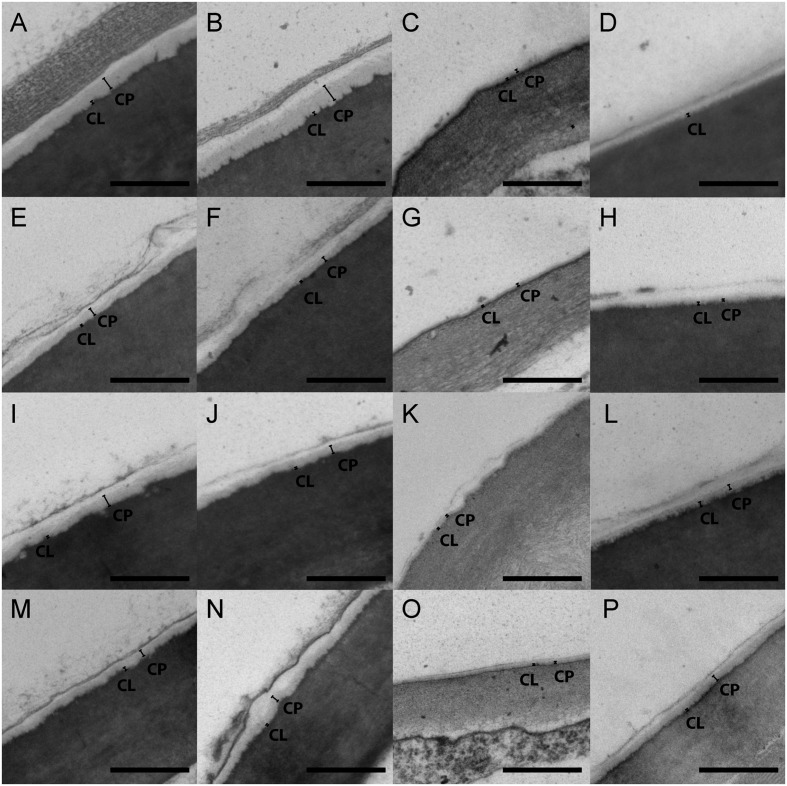
**Transmission electron micrographs of moss cuticles.** **(A–D)** *Funaria hygrometrica*. **(E–H)** *Physcomitrium pyriforme*. **(I–L)** *Physcomitrellopsis africana*. **(M–P)** *Aphanorrhegma serratum*. **(A, E, I, M)** Young calyptra. **(B, F, J, N)** Older calyptra. **(C, G, K, O)** Young sporophyte. **(D, H, L, P)** Older sporophyte. CL, cuticular layer; CP, cuticle proper. Scale bars: 500 nm.

All four Funariaceae species examined (*A. serratum, P. africana, P. pyriforme, F. hygrometrica*) have a cuticle consisting of a cuticle proper (CP) and cuticular layer (CL; **Figure 2**). These two layers are present on both the calyptra and sporophyte at all developmental stages (**Figure 2**). Epicuticular waxes were not preserved during sample preparation and thus were not quantified (**Figure 2**). Measurements were averaged to calculate a mean value for each tissue of each individual. Three individuals of each organ at both developmental stages were sectioned for each species, (young calyptrae *N* = 12, young sporophytes *N* = 12, older calyptrae *N* = 12, and older sporophytes *N* = 12).

### Morphology

Sporophyte sizes ranged from less than 1 mm tall in *A. serratum* to approximately 30 mm tall in *F. hygrometrica* (**Table 1**; **Figure 1**). Taller sporophytes were associated with larger calyptra, with calyptra ranging from less than 0.5 mm in *A. serratum* to almost 4.5 mm in *F. hygrometrica* (**Table 1**; **Figure 1**).

**Table 1:** Sizes in millimeters of calyptra and sporophytes at the two developmental stages analyzed in the study (*N* = 5 for each).

	*Aphanorrhegma serratum*		*Physcomitrellopsis africana*		*Physcomitrium pyriforme*		*Funaria hygrometrica*	
				
	Young	Older	Young	Older	Young	Older	Young	Older
Calyptra	0.35	0.28	1.0	1.0	2.2	2.4	3.7	3.8
	(0.25–0.5)	(0.25–0.33)	(1.0)	(1.25–1.5)	(2.0–2.25)	(2.25–2.5)	(3.25–4.25)	(3.25–4.25)
Sporophyte	0.60	0.75	1.7	2.2	6.0	12.1	12.9	22.9
	(0.5–0.75)	(0.75)	(1.5–2.0)	(2.0–2.25)	(5.0–7.0)	(10.0–14.5)	(11.0–15.0)	(19.0–27.0)


*Means with ranges are given in parentheses.*

### Development

**FIGURE 3:**
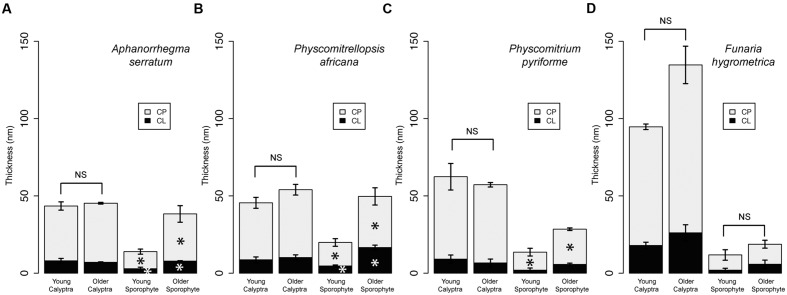
**Cuticle thicknesses for calyptrae and sporophytes at two developmental stages for four Funariaceae species.** For each developmental stage (young and older), cuticle thicknesses were measured from the periclinal cell walls of the epidermis for three individuals and then averaged (mean ±*SE*). Statistically significant differences (*P* < 0.05) between cuticle layers are starred with an asterisk. **(A)** *Aphanorrhegma serratum.* **(B)** *Physcomitrellopsis africana.* **(C)** *Physcomitrium pyriforme.* **(D)** *Funaria hygrometrica.* CL, cuticular layer; CP, cuticle proper; NS, no significant differences.

Comparisons of the cuticle layers of young and older calyptrae revealed no significant differences between the two developmental stages for the four species (**Figure 3**). Thus, in all subsequent analyses data from the young and older calyptra were combined. Significant differences in the cuticle layers were found between the young and older sporophytes for three of the four Funariaceae species (**Figure 3**). Both cuticle layers of the older sporophytes had a larger average thickness compared to the young sporophytes for *A. serratum* and *P. africana* (**Figures 3A,B**), whereas the cuticle proper was significantly different only for *P. pyriforme* (**Figure 3C**). No significant differences in the cell lumen sizes or wall thicknesses were found between young and older calyptra, whereas lumen sizes and wall thicknesses were significantly increased between the young and older sporophytes (data not shown).

### Species Comparisons

**FIGURE 4:**
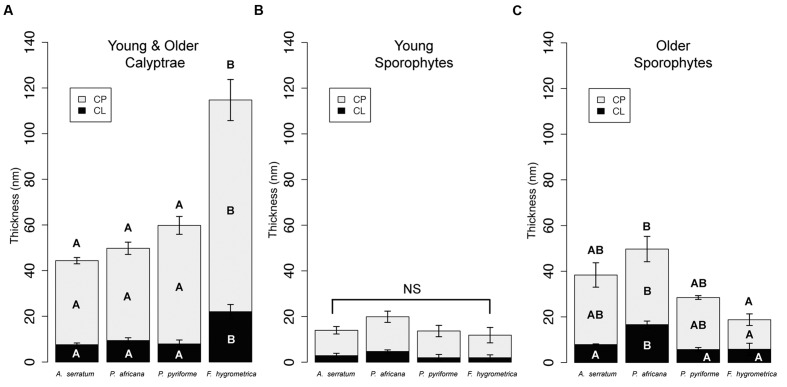
**Cuticle thicknesses for calyptrae and sporophytes for two developmental stages for four Funariaceae species.** **(A)** Calyptrae (young and older data combined). **(B)** Young sporophytes. **(C)** Older sporophytes. For each species, cuticle thicknesses were measured from the periclinal cell walls of the epidermis. Average cuticle thicknesses (mean ± SE) were calculated for six individuals per species for the calyptra (*N* = 24), combining data from young and older calyptrae, and three individuals per species for the young and older sporophytes (*N* = 24). CL, cuticular layer; CP, cuticle proper; NS, no significant differences; letters inside bars indicate layers that are significantly different based on Tukey HSD *post*-hoc tests (*P* < 0.05) for cuticle layers with significant ANOVAs.

Significant differences between species characterize the calyptra cuticle (ANOVA: CP, *F*_3,20_ = 24.97, *P* < 0.001; CL, *F*_3,20_ = 13.07, *P* < 0.001; total cuticle, *F*_3,20_ = 23.12, *P* < 0.001; **Figure 4A**). Specifically the cuticle layers of *F. hygrometrica* were significantly thicker than the cuticles of the other three species (**Figure 4A**).

Young sporophyte cuticles were not significantly different across species (**Figure 4B**), whereas the cuticles of older sporophytes were significantly different (**Figure 4C**; ANOVA: CP, *F*_3,8_ = 4.89, *P* < 0.05; CL, *F*_3,8_ = 10.23, *P* < 0.01; total cuticle, *F*_3,8_ = 6.16, *P* < 0.05). The cuticle proper of *P. africana* was significantly thicker than *F. hygrometrica*, but the other two species were not significantly different from any other species. The cuticular layer was significantly thicker on *P. africana* compared to the other three species.

### Sporophyte Height as a Proxy for Dehydration Stress

**FIGURE 5:**
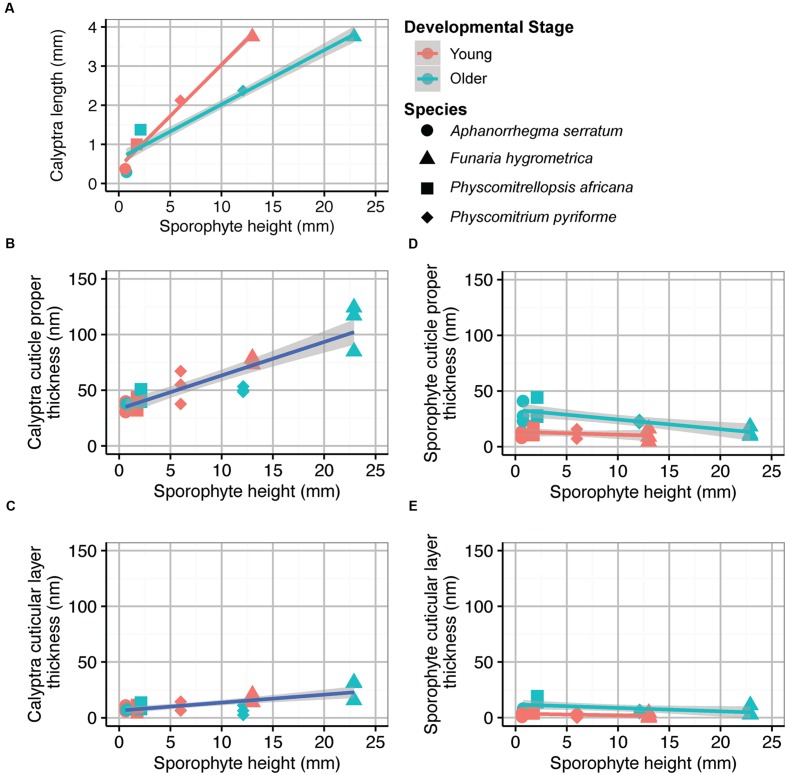
**Average organ sizes and cuticle thicknesses for four Funariaceae species (circle *Aphanorrhegma serratum*, triangle *Funaria hygrometrica*, square *Physcomitrellopsis africana*, diamond *Physcomitrium pyriforme*) at two developmental stages (young in red, older in green).** Sporophyte height and calyptra length were averaged from five individuals of each species at each developmental stage (*N* = 40). Cuticle thicknesses were measured from the periclinal cell walls of the epidermis for three individuals per species at each developmental stage (*N* = 24). All regression lines are surrounded by 95% confidence intervals in gray (young in red, old in green, combined in blue). **(A)** Sporophyte height correlated with calyptra length; Young, adjusted *R*^2^ = 0.98, *df* = 3, *P* < 0.01; Older, adjusted *R*^2^ = 0.91, *df* = 3, *P* < 0.05. **(B)** Sporophyte height correlated with calyptra cuticle proper thickness; adjusted *R*^2^ = 0.79, *df* = 23, *P* < 0.001. **(C)** Sporophyte height correlated with calyptra cuticular layer thickness; adjusted *R*^2^ = 0.48, *df* = 22, *P* < 0.001. **(D)** Sporophyte height correlated with sporophyte cuticle proper thickness; Young, adjusted *R*^2^ = -0.005, *df* = 11, *P* = 0.35; Older, adjusted *R*^2^ = 0.59, *df* = 11, *P* < 0.01. **(E)** Sporophyte height correlated with sporophyte cuticular layer thickness; Young, adjusted *R*^2^ = 0.05, *df* = 11, *P* = 0.23; Older, adjusted *R*^2^ = 0.22, *df* = 11, *P* = 0.07.

Sporophyte height and calyptra length were significantly correlated at both developmental stages (**Figure 5A**; Young, adjusted *R*^2^ = 0.98, *df* = 3, *P* < 0.01; Older, adjusted *R*^2^ = 0.91, *df* = 3, *P* < 0.05). Cuticle thickness of the maternal calyptra was also correlated with sporophyte height. Calyptra cuticle thickness data were combined from both developmental stages. The calyptra cuticle proper and cuticular layer thicknesses were significantly correlated with sporophyte height (**Figures 5B,C**; CP, adjusted *R*^2^ = 0.79, *df* = 23, *P* < 0.001; CL, adjusted *R*^2^ = 0.50, df = 23, *P* < 0.001). No significant relationships were found between sporophyte height and sporophyte cuticle thickness at both the young and older developmental stages (**Figure 5D**; CP, Young, adjusted *R^2^* = –0.005, *df* = 11, *P* = 0.35; Older, adjusted *R*^2^ = 0.59, *df* = 11, *P* < 0.01; **Figure 5E** CL, Young, adjusted *R*^2^ = 0.05, *df* = 11, *P* = 0.23; Older, adjusted *R*^2^ = 0.22, *df* = 11, *P* = 0.07).

## Discussion

The calyptra is a maternal organ covering the apex of the moss sporophyte, thereby protecting the young sporophyte offspring from dehydration at least until meiosis occurs in the apical sporangium. This structure, which is derived from the archegonium and tissues of the leafy gametophyte below, is covered by a relatively thick cuticle (Budke et al., 2011), which develops early compared to the cuticle of the underlying sporophyte (Budke et al., 2012), and it is critical for sporophyte fitness (Budke et al., 2013). These observations, based on *F. hygrometrica*, suggest that the calyptra is under strong selection. Thus, variation in the calyptra cuticle may be correlated with changes in sporophyte architecture, such that species with short sporophytes that mature surrounded by the vegetative leaves and hence within the laminar boundary layer, may be covered by a calyptra with a thinner cuticle and conversely that tall sporophytes elevating the sporangium above the laminar boundary layer prior to maturation may be protected by a thicker calyptra cuticle. We acknowledge that other aspects of the moss cuticle, including wax or cutin quantity and the ratios between waxes classes, may predict cuticular permeability (Riederer and Schreiber, 2001; Parsons et al., 2012). The present comparative study of four species provides the first insights into the relationship between cuticle thickness and sporophyte height in mosses.

The observations by Budke et al. (2012) that young sporophytes have relatively thin cuticles that do not thicken until late during capsule expansion and that the maternal gametophyte calyptra produces a mature cuticle early during sporophyte development that persists through capsule expansion are confirmed for all taxa (**Figure 3**), regardless of sporophyte size. Taller sporophytes, which likely experience higher levels of dehydration stress (Niklas, 1994), have thicker calyptra cuticles (**Figures 5B,C**). The measurements of the epidermal cell sizes and cell wall thicknesses confirm our previous developmental observations (Budke et al., 2012); the calyptra is a static structure whose cells do not significantly increase in size or develop thicker walls after detachment from the leafy gametophyte, whereas the sporophyte is undergoing dramatic changes during elongation and maturation in terms of epidermal cell size and wall thickness (data not shown). These observations are mirrored by our cuticle data, which show an early developing and then static calyptra cuticle contrasting with a dynamic, late developing sporophyte cuticle (**Figure 3**). These observations from a broader taxon sampling further support the hypothesis that the early maturing calyptra cuticle functions to protect the sporophyte apex from dehydration during early development, when moss sporophytes lack the structural protection provided by a thick cuticle (Budke et al., 2012, 2013).

Dispersing seeds and spores far away from the maternal plant both decreases resource competition and aids in the colonization of novel locations (Janzen, 1970). One way to attain larger dispersal distances is by increasing plant height (Thomson et al., 2011). In mosses, sporophyte height varies both across (e.g., Fife, 1982) and within species (e.g., Shaw, 1990). Even a small increase in sporophyte height may be enough to raise the spore filled capsule above the still air of the laminar boundary layer into more turbulent air flow, which would increase the ability of vertical updrafts to facilitate long distance dispersal events (Tackenberg, 2003). Increases in sporophyte height also increase the transpirational pull of resources from the maternal plant (Haig, 2012). Maximizing the resources an offspring acquires directly impacts sporophyte reproductive success. The potentially negative consequence is that taller sporophytes have increased exposures to dehydration stress. This is especially dangerous during the phase of stalk-building when the sensitive sporophyte apex is elevated beyond the protection of the laminar boundary layer and the protective leaves of the maternal plant. At this stage protective structures are critical for the sporophyte to avoid and ultimately survive the stress of dehydration. In mosses, taller species have both a thicker calyptra cuticle in addition to a larger calyptra (**Figure 5**). The ability to develop taller sporophytes likely shapes the efficiency of colonizing new habitats through effective spore dispersal, and thus may positively impact sporophyte reproductive success.

Tall sporophytes arise from a prolonged period of seta development, ultimately delaying capsule differentiation. During this phase, the presumptive sporangial tissues remain undifferentiated for a longer period of time compared to taxa with short and more rapidly developing sporophytes. Taller sporophytes thus have a longer period of vulnerability to damage and stress. A functional cuticle can be maintained longer either by repairing cuticle damage (Hallam, 1970; Latimer and Severson, 1997; Neinhuis et al., 2001) or by initially producing a thicker cuticle that is more resistant to damage (Onoda et al., 2012). Overall, we did not observe any significant increases or decreases in calyptra cuticle thickness across development (**Figure 3**). Though the calyptra can remain alive after detachment (True, 1906; Bopp, 1954; Oehlkers and Bopp, 1957; Wynne and Budke, 2012), it ultimately dies and may lack the resources or ability to repair cuticle damage. Alternatively, the calyptra cuticle is significantly thicker than the cuticle on all other parts of the maternal gametophyte (Budke et al., 2011; Buda et al., 2013) and we observed that species with taller sporophytes and thus longer periods of sporophyte development have thicker calyptra cuticle layers (**Figures 4A**, **5**). These observations align with a strategy of early investment in a thick calyptra cuticle that maintains its protective functions over longer periods of time by resisting damage.

Even within an individual, cuticle development is highly plastic and can be influenced by the surrounding environment (e.g., shade vs. sun leaves in *Quercus*, Osborn and Taylor, 1990; submerged vs. aerial leaves in amphibious plants, Frost-Christensen et al., 2003). The leafy gametophytes of mosses can also increase their cuticle investment in response to alternating cycles of hydration and dehydration stress (Xu et al., 2009). The ability to alter cuticle development in response to stressful conditions could be advantageous for maternal moss plants, both improving their own reproductive success and the fitness of their offspring sporophyte. Our developmental observations reported here are from plants grown in common garden conditions, thus all differences in the cuticles can be attributed solely to taxon and tissue differences, not environmental influences. The influences that shape cuticle development on the maternal calyptra specifically and in bryophytes broadly are areas ripe for exploration. Expanding our knowledge of the environmental factors that impact cuticle development will enable us to better understand the physiology and evolution of protective strategies in plants.

In bryophytes, the sporophyte remains physically attached to the maternal gametophyte throughout its lifespan. Young sporophytes do photosynthesize; however, they are dependent on nutrients and water from the maternal plant (Ligrone and Gambardella, 1988). This presents a conflict over resources between the offspring and maternal plants (Haig and Wilczek, 2006; Haig, 2012), especially for species with perennial gametophytes that will reproduce in subsequent years. The cuticle on the maternal calyptra may not only play a protective role in dehydration, but this layer may concurrently decrease sporophyte transpiration; reducing the resources taken by the offspring sporophyte from the maternal plant. On the opposite side of this conflict, the offspring potentially increases transpiration, and thus its pull of resources from the maternal plant, by increasing the number of stomata on the capsule or by increasing seta length, elevating the capsule further above the boundary layer. Our data on the calyptra cuticle directly align with the predictions of this conflict. We observed that the calyptra cuticle is thicker for species with taller sporophytes, which may enable them to limit the transpirational pull of resources by the offspring from the maternal plant (**Figure 4A**).

The evolution of sporophyte morphology across the Funariaceae is widely thought to occur via the process of reduction (McDaniel et al., 2010; Liu et al., 2012; Beike et al., 2014; Medina et al., 2015). Parallel losses in structures that facilitate spore dispersal, such as peristome teeth, the operculum, and the seta, are observed across the family. The morphological reductions observed in both the maternal calyptra and offspring sporophyte of the Funariaceae could have occurred under several alternative scenarios; driven initially by morphological evolution of either the offspring sporophyte or the maternal calyptra or alternatively evolving in concert. In one scenario the evolution of a shorter sporophyte results in lower levels of dehydration stress, enabling the maternal gametophyte to invest fewer resources in the protective calyptra, by thinning the cuticle and ultimately developing a smaller calyptra. In an alternative scenario, the evolution of a smaller calyptra with a thinner calyptra cuticle results in higher levels of sporophyte dehydration stress, constraining and ultimately reducing sporophyte height. A well-resolved phylogeny combined with comparative methods may enable us to determine the most likely scenario (Felsenstein, 1985). In either case, the cuticle represents a costly structural investment, the lipids of which may require more than double the glucose for a plant to build compared to cell wall polysaccharides (Poorter and Villar, 1997). Thus, any decrease in cuticle investment frees up resources to be allocated to other developmental, reproductive, or physiological processes.

Many maternal organisms provide protection for their offspring and this study highlights a unique example of maternal protection in plants. The maternal calyptra is not a vestigial structure, but has been retained and elaborated across the 12,500 species of mosses (Crosby et al., 1999). Using a comparative developmental framework we have expanded our knowledge of moss cuticle development to a broader number of taxa. This study lays the groundwork for future studies of morphologically and ecologically diverse species to ultimately further our understanding of the connections between maternal structures and their functional importance for offspring plants. Our observations broaden our knowledge of the plant cuticle and highlight the functionally important role the cuticle plays in preventing dehydration even in the relatively diminutive bryophytes.

## Author Contributions

JB designed, performed, and analyzed the experiments. JB and BG conceived the study and wrote the manuscript. All authors read and approved the final version of the manuscript to be published.

## Conflict of Interest Statement

The authors declare that the research was conducted in the absence of any commercial or financial relationships that could be construed as a potential conflict of interest.

## Footnotes

1 http://rsb.info.nih.gov/ij/
